# Epigenetic perspectives associated with COVID-19 infection and related cytokine storm: an updated review

**DOI:** 10.1007/s15010-023-02017-8

**Published:** 2023-03-12

**Authors:** Amit Dey, K. Vaishak, Dikshita Deka, Arun Kumar Radhakrishnan, Sujay Paul, Priyadarshini Shanmugam, Alice Peace Daniel, Surajit Pathak, Asim K. Duttaroy, Antara Banerjee

**Affiliations:** 1 Department of Medical Biotechnology, Faculty of Allied Health Sciences, Chettinad Academy of Research and Education (CARE), Chettinad Hospital and Research Institute (CHRI), Kelambakkam, Chennai, TN 603103 India; 2 Department of Pharmacology, Chettinad Hospital and Research Institute (CHRI), Chettinad Academy of Research and Education (CARE), Chennai, TN India; 3 Tecnologico de Monterrey, School of Engineering and Sciences, Campus Queretaro, Av. Epigmenio Gonzalez, No.500 Fracc., CP 76130 San Pablo, Querétaro Mexico; 4 Department of Microbiology, Chettinad Hospital and Research Institute (CHRI), Chettinad Academy of Research and Education (CARE), Chennai, TN 603103 India; 5 Department of Nutrition, Institute of Basic Medical Sciences, Faculty of Medicine, University of Oslo, Oslo, Norway

**Keywords:** SARS- CoV-2, Epigenetics, Cytokine storm, Inflammatory interleukins, Histone proteins, Prognosis

## Abstract

**Purpose:**

The COVID-19 pandemic caused by the novel Severe Acute Respiratory Syndrome Corona Virus 2 (SARS-CoV-2) has put the world in a medical crisis for the past three years; nearly 6.3 million lives have been diminished due to the virus outbreak. This review aims to update the recent findings on COVID-19 infections from an epigenetic scenario and develop future perspectives of epi-drugs to treat the disease.

**Methods:**

Original research articles and review studies related to COVID-19 were searched and analyzed from the Google Scholar/PubMed/Medline databases mainly between 2019 and 2022 to brief the recent work.

**Results:**

Numerous in-depth studies of the mechanisms used by SARS-CoV-2 have been going on to minimize the consequences of the viral outburst. Angiotensin-Converting Enzyme 2 receptors and Transmembrane serine protease 2 facilitate viral entry to the host cells. Upon internalization, it uses the host machinery to replicate viral copies and alter the downstream regulation of the normal cells, causing infection-related morbidities and mortalities. In addition, several epigenetic regulations such as DNA methylation, acetylation, histone modifications, microRNA, and other factors (age, sex, etc.) are responsible for the regulations of viral entry, its immune evasion, and cytokine responses also play a major modulatory role in COVID-19 severity, which has been discussed in detail in this review.

**Conclusion:**

Findings of epigenetic regulation of viral pathogenicity open a new window for epi-drugs as a possible therapeutical approach against COVID-19.

## Introduction

Coronavirus Disease 2019 (COVID-19) outbreak has put a worldwide emergency. It has almost stopped the mobility of the whole world, not only in the health sector but also from the economic perspective. The number of people getting infected and dying daily was skying unstoppably, and almost 6.3 million people have lost their lives due to the COVID-19 pandemic [1–3]. The new coronavirus is a severe pathogen first reported in Wuhan by the end of 2019 [4]. The World Health Organisation has proclaimed the upsurge of SARS- CoV-2/COVID-19 as a global medical crisis. The US, Italy, Spain, and the UK were severely impacted by COVID-19 infection and associated deaths in the early stages of the outbreak, and this trend continued in the second wave [5, 6]. Brazil and India, on the other hand, have witnessed high rates of infection with lower fatality rates than the countries mentioned above [7]. The severe acute respiratory syndrome coronavirus-2 (SARS-CoV-2) primarily targets the human respiratory system and displays symptoms such as cough, sneezing, high body temperature, runny nose, sore throat, anosmia, etc. for the first few days and can affect the lower airways as well accompanied by shortness in breathing, fatigue, diarrhea, vomiting, etc. [8, 9]. Many patients may experience a gradually deteriorating condition, especially those with existing medical conditions [10].

Abnormality in immune expression due to the infection may lead to other pathological conditions such as organ dysfunction, septic shock, and other pathogenic infections. SARS-CoV-2 is the RNA-based single-stranded virus that colonizes itself inside cells through any of the mucus membranes and uses Transmembrane serine protease 2 (TMPRSS2) and ACE2 receptor protein for fusion and endocytosis with the host cell. ACE2 is the primary viral receptor, hence crucial for the SARS-COV-2 pathogenicity [11]. Increased expression of ACE2 facilitates more invasion of the coronavirus inside the cells. After entering the host cell, the viral RNA undergoes translation to synthesize the viral protein and new RNA created for new virions with the assistance of RNA-dependent RNA polymerase [12, 13]. As a primary response to COVID-19 infection, the host immune system confronts a rapid upregulation of several pro-inflammatory cytokines like IL-1, IL-6, TNF- $\alpha$, and interferon in the bloodstream, often referred to as the ‘Cytokine Storm (CS)’ [14]. It causes severe inflammation, lung injury, acute respiratory distress syndrome (ARDS), and organ failure [15]. As per cytokine patterns in COVID-19 patients, the adaptive and innate immune responses activated with SARS-CoV-2 infection can lead to uncontrolled inflammatory response and proceed to CS. Epigenetic alterations, such as DNA methylation and histone tail post-translational modifications, have a role in practically all biological processes and allow cells to quick adaptation due to environmental changes by altering the conformation of genetic expression and are implicated in various human disorders. Some changes in modifying DNA-histone or RNA levels lead to severe human disorders. These changes occur when a damaged cell responds to a disease or infection to restore the typical cues [16, 17]. Patients infected with SARS- CoV-2 have shown epigenetic alterations, indicating that epigenetic pathways can be the potential targets for the therapies against viral infections [17].

Along with the host systemic responses, COVID-19 severity is also controlled by epigenetic modulations [18]. Epigenetic pathways may be altered by SARS-CoV-2, which may affect the expression of ACE2 and various immunoregulatory genes that play an important role in regulating both immune and metabolic pathways on immune and epithelial cells [19]. This will damage the tissue and augment multi-organ infections. Conventional anti-viral medications that were claimed to be useful have been repurposed. Still, the results are not convincing, and there is an unfulfilled and critical necessity for efficacious drugs, particularly against the virus [20]. The earlier epidemics caused by SARS-CoV and MERS-CoV have served as a subject for thought in the fight against the prevailing epidemic, and it shall indeed tend to aid in future SARS-CoV-2 treatments [20]. Epigenetic markers in COVID-19 and their dynamics during viral entry and throughout infection (e.g., from asymptomatic to mild symptomatic, severe infection and long persistent symptoms) can be used as a diagnostic tool and design therapeutics to control the severity of COVID-19 and related CSs [21]. Comorbidities such as Type II diabetes and cardiovascular manifestations are significant metabolic complications that contribute to the mortality of patients with COVID-19 [22]. Further insight into the epi-drug study will be helpful to have a more precise therapeutic approach to counter the fatality of SARS-CoV-2 infection [23, 24]. This review is undertaken in the purview of the importance and recent pieces of evidence on the epigenetic interplay involving SARS-CoV-2 infection to critically evaluate the aging parameters, which remain crucial for the positive outcome of the treatment strategies considering the comorbidities associated with viral infections.

## Cytokine storm and COVID-19

**Fig. 1:**
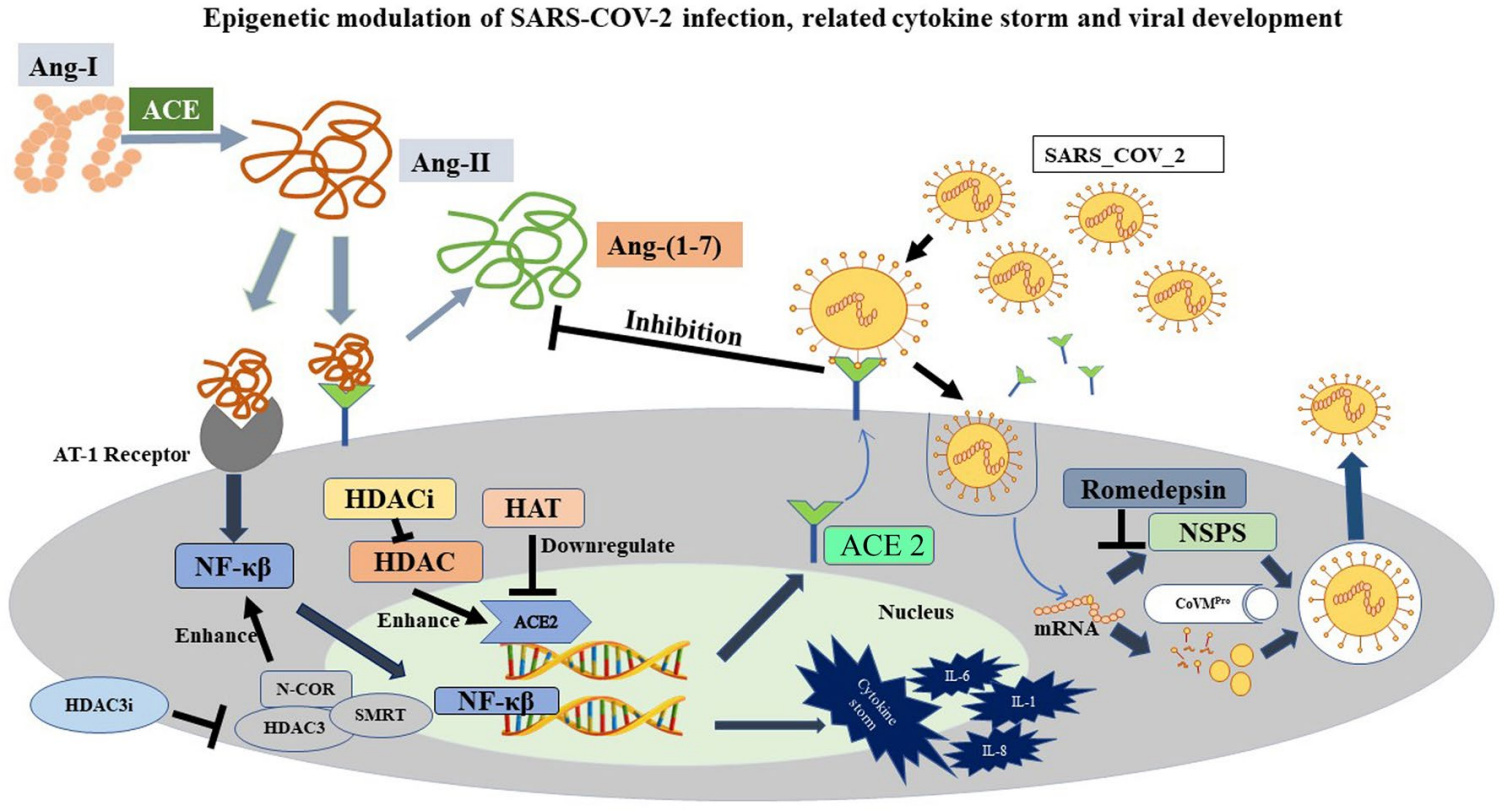
Infection of SARS-COV-2 through the airway of humans and its association with the initiation of CS

Three reported stages of disease severity could be distinguished in SARS-CoV-2 infection: mild, moderate, and severe. In the case of mild but predominantly severe COVID-19 infection, severe lung injury, multi-organ failure, and abnormal cytokine patterns often lead to the death of the patients due to CS [25–27]. The SARS-CoV-2 infection triggers an aggressive immune response in many of the patients, resulting in an excessive inflammatory response [28]. This increase in cytokines provokes an upsurge of immune cells from the vasculature, such as macrophages, neutrophils, and T cells, into the infected area, resulting in damage to human tissue due to disruption of endothelial cell–cell contacts, capillaries, and vascular barrier, alveolar tissues, along with multi-organ failure, and fatality [29–31]. A primary cause of high mortality in COVID-19 is ARDS, which results in low oxygen saturation levels [32]. Although the exact pathophysiology of ARDS in COVID-19 individuals is unknown, one of the major determining factors is the overproduction of pro-inflammatory cytokines [33, 34]. Previous research indicates that IL-1β, IL-6, IL-8, IL-12, inducible protein 10 (IP-10), MCP-1, and IFN-γ are elevated throughout SARS-CoV-2 infection [35–39]. Depleted Th2 cytokine IL-4 was also found in SARS patients [40]. Increased IL-15, IL-17, IFN- γ, and TNF levels have also been associated with MERS-CoV infection [41]. According to a few investigations, people with severe COVID-19 have elevated amounts of IL-2, IL-6, IL-7, IL-10, IP-10, MCP-1, TNF-$a$, macrophage inflammatory protein-1α(MIP-1α), and granulocyte-CSF than those with mild—to—moderate infestations [42–45]. Figure 1 describes the association of the COVID-19 virus in the initiation of CS (Fig. 1).

The severity of COVID-19 infection is highly correlated with Lymphopenia, a condition where the blood's lymphocyte level (CD8 + T cell) drastically decreases, and elevated pro-inflammatory cytokines, such as TNF-α and IL-6 [46, 47]. CS is one of the critical factors related to Lymphopenia and can alter the behavior of T-cells and NK cells [48–50].

Hemodynamic instability, systemic inflammation, multi-organ failure, and hyperferritinemia are CS's main signatures, which can deteriorate a patient’s condition and lead to death [51]. The CS-related complexities are caused by the abnormal aberration of pro-inflammatory cytokines IL-1, IL-6, IL-18, IFN-$\gamma$, and TNF-$\alpha$ that has been reported in the studies of influenza H5N1, influenza H1N1 and two coronaviruses- ‘Severe acute respiratory syndrome (SARS) associated coronavirus’ (outbreak in February, 2003, around five countries including China)[52] and ‘Middle East respiratory syndrome coronavirus(MERS-CoV)’, closely linked to COVID-19 [53, 54]. IL-6 and TNF- $\alpha$ are the major players in the CS's interplay[15]. Patients acquire ARDS, leading to acute lung damage that can cause mortality without immediate intervention. Therefore, CS is one of the major health concerns for COVID-19 patients, as negligence may risk the patient’s life [55]. To cope with the severity of the COVID-19, Clinicians are using seven primary therapeutic strategies including the use of anti inflammatory drugs, anti-viral and anti thrombotic drugs, therapies for Acute-Hypoxamic-respiratory-failure, use of anti SARS-CoV-2 antibodies, drugs for Renin-angeotensin-Aldosterone system modulation and vitamins [56]. Anti-inflammatory medicines that reduce cytokine responses are believed to reduce morbidity and death in infected patients, as well as anti-viral therapies that deliberately target the virus [57]. Severe Covid patient often suffer from COVID-19-associated-Coagulopathy, hence required to take antithrombotic therapies [58].Timely screening of CS and rapid treatment can lead to a better prognosis [59]. Biological medicines that address cytokines have indeed been suggested as therapies for CS. IFN-α2b administration, combined with Arbidol, can accelerate the clearance of the virus and reduce the IL-6 and CRP levels to normal [60]. Glycyrrhizic Acid is a potent inhibitor of CS. Studies have shown it can inhibit IL-33, which plays a key role in ARDS. It also inhibits the production of IL-1β, IL-6, IL-8, and TNF-α production, which are the major player in the CS [61]. Anti-viral therapies like use of Remdesivir, Nirmatrelvir–ritonavir, Molnupiravir are recently inducted into practice with excilent outcomes [56]. Adalimumab and infliximab are two anti-TNF antibodies that target TNF-α [62]. Elevation in pro-inflammatory cytokine levels increases the severity of the disease and mortality as well. FDA-approved Topoisomerase-1 (TOP-1) inhibitor topotecan (TPT) subdues SARS-CoV-2-induced overproduction of inflammatory cytokines in infection-induced inflammation in hamsters [63]. Apart from these, Anakinra and Tocilizumab are two of the drugs that have been studied enormously. Anakinra, an IL-1 receptor antagonist often used to treat rheumatoid arthritis, was reported to be beneficial in the therapies of cytophagic histiocytic panniculitis with subsequent hemophagocytic lymphohistiocytosis, an illness associated with drastic CS [64]. Tocilizumab is a recombinant humanistic IL-6 potent inhibitor that blocks IL-6 from linking to its receptor, interrupting signaling [65]. Tocilizumab treats juvenile idiopathic arthritis, rheumatoid arthritis, giant cell arteritis, and several types of acute inflammatory diseases, including CS caused by Chimeric antigen receptor (CAR-T) cell treatment for hematological malignancies. In a clinical study including 15 COVID-19-positive patients, tocilizumab treatment was found to significantly lower C-reactive Protein (CRP) levels. [66]. It was considered in a randomized clinical study as a possible treatment for COVID-19-associated pneumonia and high CRP levels [67]. JAK and other downstream cytokine inhibitors are also being investigated as possible CS treatments [68]. Baricitinib, an example of JAK 1/2 inhibitors, is the first immunomodulating drug approved by the FDA(U.S) [69]. There were encouraging findings in clinical research where 15 patients with moderate to severe COVID-19 infection were given baricitinib with hydroxychloroquine, and 11 of them recovered [70]. It is also reported to improve the mortality rate effectively [71]. A new study should focus on therapeutic approaches to manage COVID-19-related CS to lower COVID-19-related death rates. Apart from these therapeutic drugs, corticosteroids such as dexamethasone, anti-viral drugs, and hydroxychloroquine have been used as therapeutic options to treat severe COVID-19 infection accompanied by CS. However, glucocorticoids and mineralocorticoids have effectively managed CS in critically ill patients with COVID-19, controversy over their effectiveness has been reported in patients with pneumonia [72]. Neutrophil Extracellular Trape (NET) is a natural defense mechanism of the human body that gets activated upon the invasion of pathogens. It has the interplay of many enzymes, such as neutrophil elastase (NE), peptidyl arginine deiminase type 4 (PADA4), etc., that cause an explosion of neutrophil cellular content that eliminates the pathogens. In the case of COVID-19, the neutrophil level increases with Lymphopenia significantly. CS and downregulation of ACE2 receptors activate the NET, which along with the pathogen, collaterally damages the vascular endothelium and lung epithelium which deteriorate the condition further. Prostaglandins, thrombomodulin, activated protein C (APC), anti-high mobility group box-1 (HMGB-1), and heparin are some of the endogenous molecules that inhibit NET activation. Whereas aspirin, sivelestat, and cyclosporine are some of the exogenous drugs used for the same purpose [73].

## Epigenetic modulations in COVID-19 infection

Epigenetics is a branch of biology describing the genetic expressivity in response to environmental cues, such as food, temperature, humidity, pollution, etc., and the expression of other phenotypic traits, such as age and sex [74–76]. Modifying DNA conformation inside the chromosome gives access to the transcription factors or denies it, which is the fundamentals of epigenetics. Several epigenetic modifications, such as DNA methylation, histone methylation, acetylation, deacetylation, and telomere shortening, play a crucial role in the COVID-19 pathophysiology [77]. The host’s cellular receptors act as the recognition site for the viral spike (S) protein and work as the viral entry point into the host cell. In the case of SARS-CoV-2, one of the significant cellular receptors that facilitate viral inclusion is the Angiotensin-converting enzyme 2 (ACE2) [78]. The S protein of SARS-CoV-2 has two subunits—S_1_ and S_2_. S_1_ binds to ACE2, and S_2_ mediates the fusion of the virus with host cells [79]. Recent publications have confirmed that overexpression of ACE2 is associated with enhanced severity and susceptibility to the disease [80–82]. ACE2 converts Angiotensin-II (Ang II), a product of Ang-I conversion by ACE, into Ang-(1–7) in a normal human [83]. In a COVID patient, internalization of the virus into the cells exfoliates the ACE2 and reduces its expression on cell membranes [84]. This, in turn, increases the level of Ang-II and leads to the overproduction of cytokines like IFN-$\gamma ,$ IL-6, TNF-$\beta$ etc. [85, 86] through JAK/STAT pathway and by inducing the Nuclear Factor kappa-B (NF*κ-*B), eventually causing CS in patients and deteriorate their condition [87]. The accumulation of Ang-II can hyperactivate the Angiotensin-II type -1 receptor (AT_1_R) and increase pulmonary capillary permeability, causing pulmonary edema [88]. Comorbidities can worsen the situation by increasing the Ang-II level further. For example, a COVID patient with type two diabetes has a higher blood IL-1 $\beta$ concentration, which increases the expression of ACE by elevating the expression of Hypoxia Inducing Factor-1 $\alpha$ (HIF-1 $\alpha$) [89]. Several epigenetic conditions such as food, smoking habit, etc. have a role in controlling the expression of ACE2 [90]. Mostly male, aged, and smokers show hypomethylation in the ACE2 gene and therefore, overexpression of ACE2 makes them more vulnerable to COVID. On the other hand, Women, children and nonsmokers show hypermethylated ACE2 gene; consequently, they are less susceptible to the disease [91]. According to some studies, consuming polyunsaturated fatty acids increase the expression of ACE2 and A Disintegrin and Metalloprotease 17 (ADAM 17) [92, 93]. Concurrent expression of both these genes reduces the expression of ACE2 on the cell surface. Many epigenetic processes, such as DNA methylation, telomere shortening, and especially DNA acetylation, are responsible for the expression and control of the *ACE2* gene [94].

ABO blood grouping antigen is also getting the spotlight as a parameter of COVID-19 severity. Several studies show evidence that ‘non-O’ individuals are at higher risk than individuals with the O blood group. However, the exact reason is still unknown [95, 96].

### DNA methylation

Methylation of CpG island through DNA Methyl Transferases (DNMTs) silent the gene expression. Methylation at the promoter region prevents the binding of transcription factors and results in transcriptional inactivity. On the other hand, DNA demethylase removes the methyl groups and allows genes to express [97]. Hypomethylation of two CpG regions (cg16734967 and cg23232263) at the ACE2 promoter region of human lung tissue significantly differs in males and females, where females were found with more expressivity of the gene than males. ACE2 promoter methylation status in uterine corpus endometrial cancer and renal papillary cell carcinoma tissues are deficient, making them more susceptible to SARS-CoV-2. Similarly, Chronic Obstructive Pulmonary Disease (COPD) and smoking habits in the patient show CpG hypomethylation making the individual more prone to COVID [98]. COVID-19 infection alters the methylation pattern in the CpG site of interferon-related genes and antigen-presenting genes, causing changes in gene expression. Inflammatory cytokines influence DNA methylation alteration during myeloid differentiation [99]. When COVID-19-infected patients are compared with normal individuals, a hike in the hypomethylated signals of interferon-inducible genes and enrichment of hypermethylation signal of ‘FC Gamma Receptor dependent phagocytosis (FCGR phagocytosis)’ related genes have been observed [100]. ‘Interferon Alpha inducible protein 27 (IFI27)’, a known biomarker gene for influenza infection, is reported to be hypo methylated during COVID-19 infection, which alters the innate immune response upon viral infection. Similarly, ‘Epithelial Stromal Interaction 1 (EPSI1), important for macrophage differentiation, gets hypomethylated at particular CpG sequences in COVID-19 patients. Interferon Regulatory Factor 7 (IRF7), which plays an essential role in innate immunity, is less methylated at particular CpG sites of COVID-19 patients than the normal individual [101]. The DNA methylation status of other genes also influences the circumstances of COVID patients. For example, syncytin 1 and 2 are the two genes responsible for syncytium formation during placental development. SARS-CoV-2 also uses the same genes to facilitate syncytium formation to enter the host cells and multiplicate. Generally, in tissues other than the placenta, these two genes are found to be hypermethylated. But in the case of viral infection, the genes become hypomethylated and facilitate the inclusion of the viral particles into the host cells [102, 103].

### Histone modifications

Histone modification is another modulator of epigenetic regulation of COVID severity. Adding an acetyl group to the positively charged lysine residue neutralizes the overall positive charge of histone and allows the access of transcription factors to the genes [104]. Histone acetylation and deacetylation work as the molecular switch to turn on and off the expression of genes and two types of enzymes which majorly play a role are Histone Acetyl Transferase (HAT) and Histone Deacetylase (HDAC) [105]. Histone lysine acetylation activates the expression of ACE2 receptors in humans. Hyperacetylation in histone 3 (H3AC) increases the H3K4 methylation [106]. Several positively associated genes, along with ACE2, are also regulated by H3K27 acetylation. Studies have reported that HDAC can contribute to SARS-CoV-2 pathogenicity in several ways – i) HDAC upregulates ACE2 expression and promotes viral entry to the cells [107], ii) HDAC activates pro-inflammatory responses against viral infections and may give rise to CS [108] iii) HDAC activity accumulates Acetyle Co-A, which elevates the cholesterol level [109]. Increased cholesterol levels can promote viral entry to the cells. In stressed conditions, NAD-dependent HDAC Sirtuin-1(SIRT1) regulates the expression of ACE2. Studies on SARS-CoV-2-infected patients revealed that a higher transcription rate of ACE2 can be stimulated by SIRT1 [110]. Even histone deacetylation may induce pulmonary fibroblast formation in COVID-19 survivors by altering the TGF-$\beta$ signaling and ERK/PI3K pathway [111]. HDAC7 plays a significant role in TGF-$\beta$ mediated fibroblast formation, which may cause mortality in COVID-19 patients. Alteration in TGF- $\beta$ expression may lead to the over expression of cytokines such as IL-7 and increase the severity [112]. HDAC8 also induces fibroblast myofibroblast differentiation in Idiopathic Pulmonary Fibrosis [113, 114]. According to studies, corticosteroids can downregulate inflammatory gene expression by inhibiting HAT and recruitment of HDAC2 [115]. Unlike other HDACs, the downregulation of inflammatory genes through HDAC2 reduces the chances of CS [116]. HDAC3 forms a multiprotein complex with a silencing mediator for retinoid and thyroid receptor (SMRT), nuclear receptor corepressor (NCOR). It suppresses the NF-K $\beta$ activation by deacetylation of p65, which upregulates inflammatory genes such as IL-6, IL-1 $\beta$, TNF-$\alpha$ etc. and can be a potential modulator of the CS [117]. HDAC1 also is a regulator of NF-k $\beta$ inactivity. Phosphorylation of HDAC3 by CK2 and HIPK2 can be a potential regulator of the cytokines storm. A high cholesterol diet (HCD) reduces the acetylation in the ACE2 promoter region and increases the susceptibility of SARS- CoV-2 infiltration inside cells [94]. High-fat-fed mice treated with rh ACE2 shows a higher level of H3K9 acetylation [118]. HDAC interventions are also found in viral trafficking via deacetylated microtubule and can be another aspect of COVID-19 experiments. Lysin demethylase KDM5B de- methylates H3K4me3 trimethylated Histone 3 residues which reduce the expression of mir-125a, followed by the upregulation of the ACE2. Hypoxic condition is known to alter the functionality of several demethylases and therefore change the expression of the gene [119, 120]

### Histone deacetylase inhibitors (HDACIs)

**Fig. 2:**
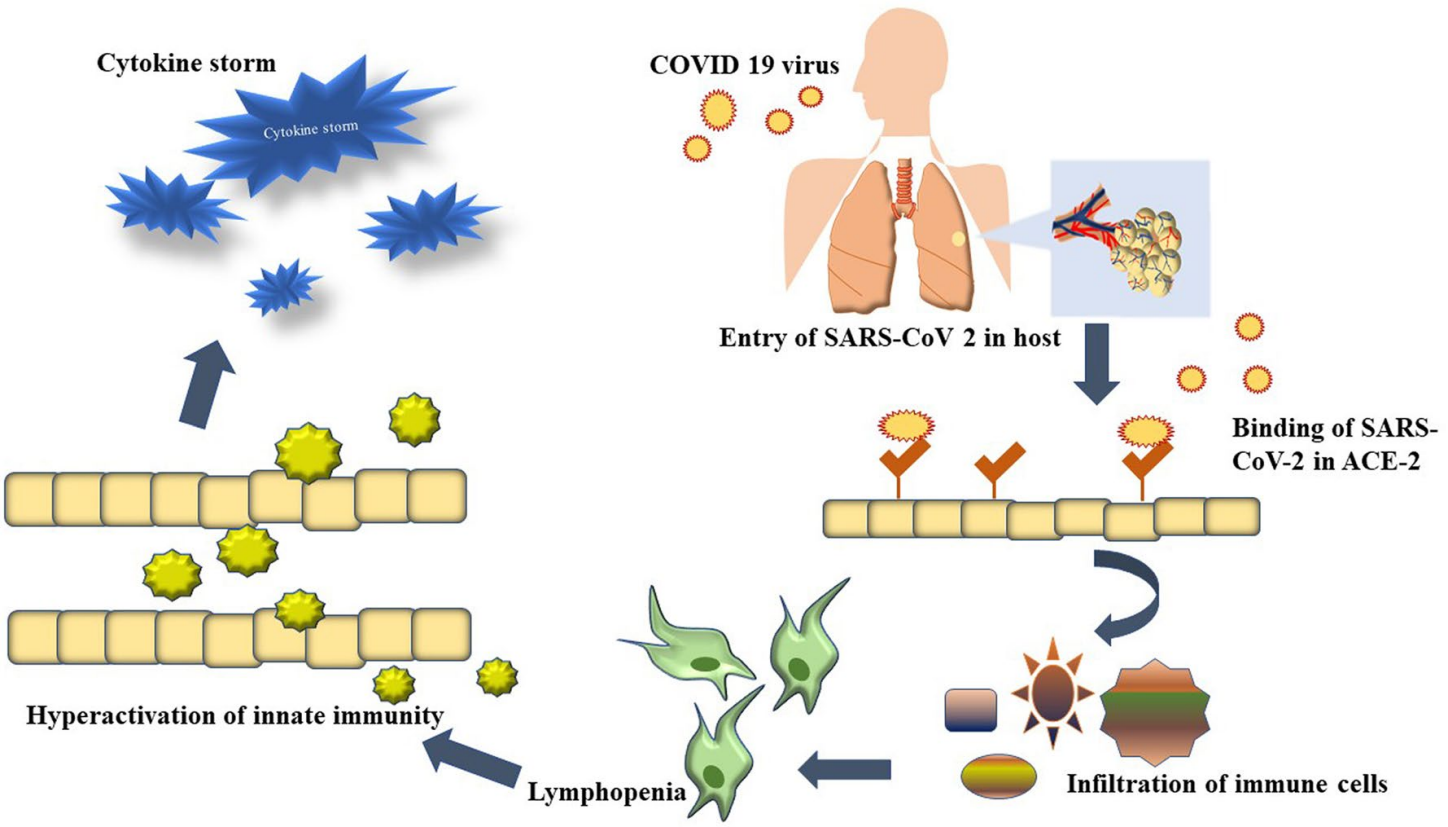
Epigenetic modulation of SARS- COV-2 infected cells and development of CSs with potential inhibitors controlling the down streaming regulations

As histone deacetylation plays an important role in the expression of many genes responsible for viral inclusions, several histone deacetylase inhibitors have been reported to downregulate ACE2 expression, making it a potential modulator of COVID-19 and related CS. Saiz et al., in their *in-vitro* study, have shown that HDAC inhibitor Valproic acid (VPA) reduces ACE2 expression significantly [121] and Neuropilin-1 (NRP_1_) on the cell surface, and the effect remains post-infection of SARS-CoV-2 [122]. Moreover, it reduces the inflammatory Cytokine’s expression and production of infectious SARS-CoV-2 virus in a dose-dependent manner. Romidepsin (drug ID: XJY-3), an HDAC inhibitor, has been reported to significantly limit the entry of SARS- CoV-2 in a clinical *in-vitro* investigation using nine FDA-approved medications to block the entry of pseudotyped SARS- CoV-2 [123]. The results suggest that HDAC inhibitors indirectly regulate viral inclusion in the host cells through SARS-CoV-2 host protein–protein interaction, directly influencing the ACE2 function. The invasion of SARS-CoV-2 inside neuronal cells provokes neurological damage [124]. During CS, the blood–brain barrier may be destroyed in COVID-19 patients and cause ischemic, hemorrhagic strokes [125]. HDAC inhibitors show neuroprotective effects and downregulate the pro-inflammatory genes [126]. Molecular docking of HDAC inhibitors against COVID-19 shows Romidepsin and its active form (RedFK) have great potential to bind to the binding site of viral protease CoVM^pro^ and block its activity [127]. It stops the virus from entering the host cells. Selective inhibition of HDAC6 reduces cytokine release by airway epithelial cells, monocytes, and macrophages. HDAC inhibitors hinder the expression of INF-1 in both airway epithelial and immune cells, which helps counter the critical conditions of COVID-19 patients. Another experiment regarding the effect of HDAC inhibitors on suppressing ACE2, ABO blood antigen, and TMPRSS2 expression has revealed that cells treated with Sodium Butyrate or Panobinostat suppress the expression of ABO and ACE2 expression but not suppress TMPRSS2 [128]. Therefore, HDAC inhibitors like panobinostat and sodium butyrate can potentially treat COVID-19. Further, the MirNet study has reported that HDAC inhibition can reduce the expression of ACE2, followed by the reduction of SAR-COV-2 infectivity [129]. Figure 2 describes the epigenetic modulation of SARS- CoV-2 and related CSs (Fig. 2).

## Epigenetic basis of viral immune evasion

Previous studies have highlighted that the hypomethylation of the ACE2 gene is associated with the severity of systemic lupus erythematosus patients after confronting SARS- CoV-2 infection with peripheral blood T-cells. TNF-α enhances DNA methylation in ACE promoters by decreasing the activity of DNMTs and regulating the expression of ACE2. DNMTs, TET1, MAX, KDM5, and HDAC2, are pivotal in regulating ACE promoter methylation with the viral entry into the cells [108]. After entering the host cell, viral positive RNA strands get localized inside the cytoplasm, using the host machinery and synthesizing a new complementary negative strand. Negative strands then create more copies of positive strands and scale up the production of the viral proteins, such as NSP-5/13/14/16, IDP or IDR, E-protein, etc. In addition, NSP5 interacted with both t-RNA methyl transferase-1 (tRMT1) and HDAC2 and restricted them outside the nucleus. Both genes are vital in inflammation regulation after infections [130].

The affinity purification-based mass spectrometry has shown that SARS- CoV-2 viral protein ‘E- protein’ shows structural similarity with histone H2a and similar affinity with bromodomain 2&4 (BRD 2/4). Therefore, after infection, it alters the binding of BRD (2/4) with HS2 inside host cells, altering the expression of several immunological pathways and allowing immune evasion of the virus. Bromodomain is a chromatin-associated protein regulating chromatin-based gene transcriptions [131].

Post-infection expression of interferon-stimulated genes (ISGs) allows the binding of transcription factors such as STAT1 and IRF 7 and allows downstream immune modulation. For example, H3K4 me3 activation induces chromatin relaxation and allows transcription factors to bind, whereas H3K27me3 promotes the chromatin's condensation and suppresses the gene's expression. After COVID-19 infection, type I and III interferons (INF) activate H3K4me3 and allow ISG expression [132].

**Fig. 3:**
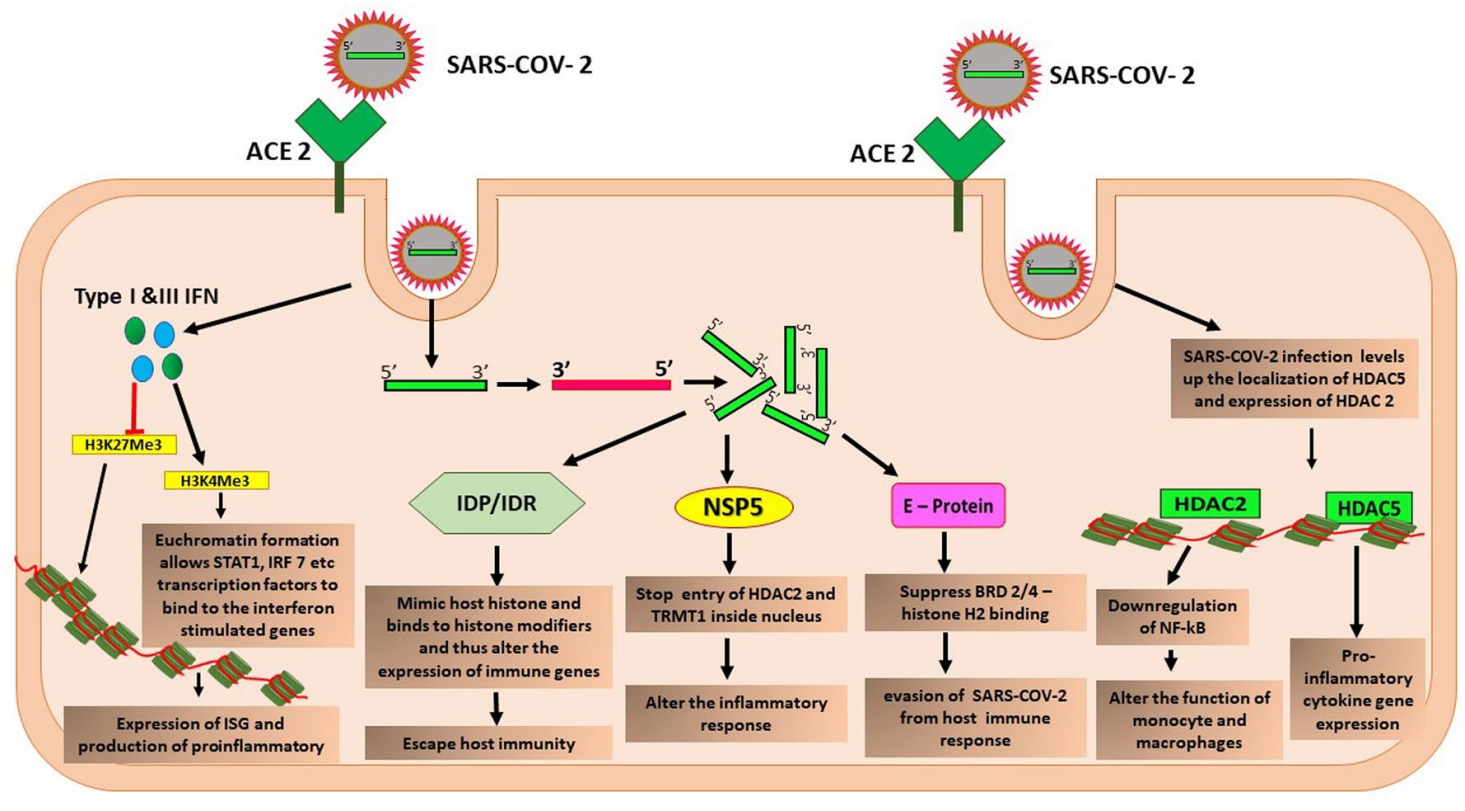
Epigenetic manipulation of host cells by SARS-COV-2 facilitates immune evasion

Viral structural proteins containing IDP (intrinsically disordered protein) or IDR (intrinsically disordered region) lack 3D shape in native conformation. It mimics eukaryotic short linear motifs (SLiMs) responsible for maintaining the host defense strategy. SLiMs are also a part of histones, which plays their role as a target for the histone modifiers. Viral IDP/IDR mimics the targets and does not allow histone modification in host cells, therefore altering the anti-viral responses and facilitating immune evasion of the virus [133]. The protein encoded by the ORF8 gene of SARS-CoV-2 mimics the ARKS motifs of H3, disrupts the epigenetic regulation, impedes the post-translational modification of the histone, and promotes chromatin compaction [134]. In SARS-CoV-2 infected cells, HDAC5 has been reported to be localized inside the nucleus, where it is found to produce pro-inflammatory cytokines and regulates the response to inflammation. In response to SARS-CoV-2 infection, HDAC2 overexpression has been noticed and found to inhibit NF-κB activity, altering monocyte and macrophage function and modulating host cell response [108] (Fig. 3).

## Interactions between human epigenetic factors and SARS-CoV-2 proteins

According to a study, 332 human proteins, some of which are epigenetic regulators, strongly interact with the SARS-CoV-2 proteins. Any alteration in these epigenetic enzymes or proteins acknowledges alteration of normal cellular function and elevates the disease conditions. The viral proteins have been identified to associate with human epigenetic factors. In recent affinity purification mass spectrometry (AP-MS) study, 26 viral proteins were mapped along with human proteins and epigenetic modifiers. Eight of the several modifiers, HDAC2, BRD2/4, CUL2, etc., have shown potent interaction with viral proteins such as NSP5, E- protein, ORF10, etc. [135]. Studies have indicated that HDAC2 has a cleavage site between the deacetylase domain and its nuclear localization motif, allowing NSP5 to reorder the enzyme and obstruct the downstream inflammatory responses. HDAC2 deacetylates H4K16 at ISG promoters for optimal ISG expression, but its association with NOS1 prevents HDAC2 from inducing the inflammatory response [136]. It was discovered that the viral E protein interacts with BRD2 and BRD4, the two key proteins to modify histones to activate transcription. The N-terminal portion of histone H3, which interacts with BRD proteins, is most likely replicated by the C-terminal area of E proteins, resulting in a contract between E and BRD proteins [137]. ORF10, a SARS- CoV-2 protein, interacts with components of the human Cullin-RING E3 ubiquitin ligase complex RBX1, ELOB, ELOC, CUL2, and ZYG11B. The complex ubiquitinates proteins so that the 26S proteasome can degrade them. ORF10 is thought to bind CUL2 to enhance viral replication by hijacking CUL2-mediated ubiquitination and destruction [138]. Several proteins with epigenetic functions linked to SARS-CoV-2 infection have kinase activity, which can be targeted using kinase inhibitors. Imatinib, a medication that has been identified as a potential treatment for SARS-CoV-2 and SARS-CoV, is currently being tested in COVID-19 clinical trials (NCT04346147, NCT04357613, NCT04422678, and NCT04394416). Quercetin is a plant-derived substance with anti-inflammatory and anti-viral properties that have been studied as a dietary supplement or COVID-19 prophylaxis in clinical studies [139]. Further, an assessment of the SARS- CoV-2 interactome proved that several viral proteins (nsp5, nsp8, nsp13, E) engage with epigenetic and gene expression regulators [140].

## Role of miRNAs in SARS-CoV-2 prognosis

A regulatory triangle is created among the host and the virus, viral-encoded miRNAs, host miRNAs, and both mRNA and miRNA targets, controlling the severity of the infection. In older individuals, the less abundance of the host defense miRNAs might explain the severity of COVID-19 and lead to death. In the host miRNA profiling of 67 SARS-CoV-2 patients from 24 countries worldwide, Khan et al. discovered that induced mi RNA either neutralizes the viral expression or acts as a proviral factor. The study has stated miRNA can be a potential therapeutical option against COVID-19 complications [141]. The viral miRNAs are inhibitory to the anti-viral proteins produced by the host, increasing the chances of SARS- CoV-2 infection by altering Janus kinase (JAK) 1 and 2 as well as signal transducer and activator of transcription (STAT) 3, 4, 5B, and 6 cellular genes and by suppression of the cytokine signaling (SOCS) cellular genes. For example, hsa-let-7a, hsa-miR-129, hsa-miR-125a-5p, hsa-miR-101, hsa-miR378, hsa-miR23b, hsa -miR, hsa-miR380-5, and hsa-miR, may target the virus [142]. Based on the gene ontology nucleotide similarity, another study analyzed miRNAs from five SARS- CoV-2 genomes and identified 22 potential viral miRNAs linked to 12 human miRNAs [143], where the interaction between human miRNAs with the viral genome may affect the host pathways that are uncertain about the virus pathogenic conditions [90].

## Discussion

SARS-CoV-2 has kept the whole world to a halt since its outbreak, not only for health but also economically. Though the discovery of several vaccines fast-tracks the recovery from the crisis, it is still a major priority to find the proper therapeutic approach to deal with the critical conditions of such viral epidemics and new alternative strategies to attenuate viral pathogenicity as well as to modulate the post-infection abnormal immune response need to be mapped out. Epigenetic pathways show the pathophysiological possibilities of COVID-19 and can be a potent player in therapeutics against viral infections [144]. In our review, we have already mentioned several studies where epigenetic modifications have successfully reduced the severity of SARS-CoV-2 pathogenicity [145]. In contrast, many ongoing experiments are yet to be reported. The CS, causing widespread tissue damage resulting in multi-organ failure and death to the patient, is also regulated through epigenetic modulation [146]. The role of DNA methylation, histone acetylation deacetylation, and other epigenetic pathways can be potential targets for the therapeutics of COVID-19. HDAC inhibitors are already being in the study and found to be successful against the disease, and many of them are approved run-in-the-mill drugs. Whether in cell culture or mouse model, epigenetic drugs are the potential candidates acting as prominent anti-viral drugs. A recent study based on computational biology analysis reported that plant-based medicines like Calanolides A, Holy Basil, Kuwanon-L, and Patentiflorin A could be regarded as potential anti-HIV viral medicines which can also be administered against SARS-CoV-2. Some studies highlighted that inhibitory machinery blocking the binding of host cell receptors to viruses and inhibiting the cellular entry of viral protein could be an effective therapeutic target [147, 148]. However, experimental results are yet to be done to validate the above-mentioned therapeutic options.

## Conclusion and future perspectives:

More recently, the HDAC inhibitors like VPA, panobinostat, Romidepsin, and vorinostat have been combined with antiretroviral therapy. According to the studies, inhibition of the BRD2 gene can also downregulate the SARS- CoV-2 pathogenicity. One such potent inhibitor is the dual bromodomain BET inhibitor. However, this attribute is limited by the incomplete latency reversal or insufficient clearance of latency-reactivated cells, which further seeks immune enhancement treatments [149–151]. Epigenetic expressions can also be used to assess the severity of the patient's situation and diagnose the infection. For example – DNA methylation-based approach for diagnosing the COVID-19 infection and its severity, named EPICOVID, is used to check the expression of the epigenetic signatures [152]. Additionally, anti-NET therapy from either synthetic or natural sources may mitigate SARS-CoV-2 infection-induced exaggerated hyperinflammation. Combinational therapy, including immunomodulators and IL-6 blockers like tocilizumab/baricitinib, and remdesivir, also improved the respiratory status of COVID-19 patients. Epigenetic signatures of COVID-19, related comorbidities, and different phases of infections (e.g., from asymptomatic to mild symptomatic, severe infection and long persistent symptoms) can be useful tools for timely diagnosis and designing therapies that may reduce the severity of COVID-19 and related fatalities. Further investigation focusing on epi-drugs can give us a closer look at possible pathways for therapeutical approaches against viral outbreaks.

## Data Availability

Not applicable.

## Abbreviations

COVID-19: Coronavirus disease 2019
ACE2: Angiotensin-converting enzyme 2
SARS-COV-2: Severe acute respiratory syndrome coronavirus-2
ARDS: Acute respiratory distress syndrome
TOP I: Topoisomerase I
CS: Cytokine storm
MERS-CoV: Middle east respiratory syndrome
TNF α: Tumor necrosis factor
MCP: Monocyte chemotactic protein
CRP: C Reactive protein
TPT: Triose phosphate transmembrane
JAK: Janus kinase
CAR-T: Chimeric antigen receptor T cell therapy
NET: Neutrophil extracellular trape
NE: Neutrophil elastase
PADA: Peptidyl arginine deaminase
APC: Activated protein C
HMGB-1: High mobility group box 1
NF-kβ: Nuclear factor- kappa β
ATR: Angiotensin receptor
HIF-1: Hypoxia inducing factor
ADAM 17: ACE2 and a disintegrin and metalloprotease 17
DNMT: DNA methyl transferase
COPD: Chronic obstructive pulmonary disease
KDM: Lysine demethylase
HAT: Histone acetyl transferase
HDAC: Histone deacetylase
H3AC: Hyper acetylation histone 3
SIRT1: Sirtuin 1
SMRT: Silencing mediator for retinoid and thyroid recptor
NCOR: Nuclear receptor corepressor
VPA: Valproic acid
NRP: Neuropilin
TMPRSS2: Transmembrane serine protease 2
TET1: Tet Methylcytosine dioxygenase 1
MAX: MYC Associated factor X
tRMT1: TRNA Methyltransferase 1
BRD: Bromodomain
ISG: Interferon stimulated genes
STAT: Statin
IRF: Iron regulatory protein
IDP: Intrinsically disordered protein
IDR: Intrinsically disordered region
SLiM: Short linear motif
AP-MS: Affinity purification mass spectroscopy
CUL: Cullin
NSP5: Non structural protein 5
RBX1: RING box 1
ELOB: Elongin B
ELOC: Elongin C
ZYG11B: Zyg-11 family member B
SOCS: Suppression of cytokine signalling
